# Investigating the association between ethnicity and health outcomes in SARS-CoV-2 in a London secondary care population

**DOI:** 10.1371/journal.pone.0240960

**Published:** 2020-10-28

**Authors:** Aatish Patel, Ahmed Abdulaal, David Ariyanayagam, Kieran Killington, Sarah J. Denny, Nabeela Mughal, Stephen Hughes, Nupur Goel, Gary W. Davies, Luke S. P. Moore, Esmita Charani

**Affiliations:** 1 Chelsea and Westminster NHS Foundation Trust, London, United Kingdom; 2 Imperial College London, Kensington, London, United Kingdom; 3 North West London Pathology, Imperial College Healthcare NHS Trust, London, United Kingdom; 4 Imperial College London, NIHR Health Protection Research Unit in Healthcare Associated Infections and Antimicrobial Resistance, Hammersmith Campus, London, United Kingdom; Universidad del Desarrollo, CHILE

## Abstract

**Background:**

Black, Asian and minority ethnic (BAME) populations are emerging as a vulnerable group in the severe acute respiratory syndrome coronavirus disease (SARS-CoV-2) pandemic. We investigated the relationship between ethnicity and health outcomes in SARS-CoV-2.

**Methods and findings:**

We conducted a retrospective, observational analysis of SARS-CoV-2 patients across two London teaching hospitals during March 1 –April 30, 2020. Routinely collected clinical data were extracted and analysed for 645 patients who met the study inclusion criteria. Within this hospitalised cohort, the BAME population were younger relative to the white population (61.70 years, 95% CI 59.70–63.73 versus 69.3 years, 95% CI 67.17–71.43, p<0.001). When adjusted for age, sex and comorbidity, ethnicity was not a predictor for ICU admission. The mean age at death was lower in the BAME population compared to the white population (71.44 years, 95% CI 69.90–72.90 versus, 77.40 years, 95% CI 76.1–78.70 respectively, p<0.001). When adjusted for age, sex and comorbidities, Asian patients had higher odds of death (OR 1.99: 95% CI 1.22–3.25, p<0.006).

**Conclusions:**

BAME patients were more likely to be admitted younger, and to die at a younger age with SARS-CoV-2. Within the BAME cohort, Asian patients were more likely to die but despite this, there was no difference in rates of admission to ICU. The reasons for these disparities are not fully understood and need to be addressed. Investigating ethnicity as a clinical risk factor remains a high public health priority. Studies that consider ethnicity as part of the wider socio-cultural determinant of health are urgently needed.

## Introduction

Ethnicity as a determinant of health has come to the fore as we collectively experience the global health pandemic caused by the severe acute respiratory syndrome coronavirus disease (SARS-CoV-2). Black, Asian and minority ethnic (BAME) populations are emerging as a vulnerable group in this pandemic [1–4]. Ethnic inequalities in SARS-CoV-2 have been reported in the United Kingdom (UK) with BAME patients at up to three times higher risk of mortality when compared to white patients [1].The data from intensive care units in the UK indicate up to one third of SARS-CoV-2 patient admissions to be from BAME populations [5]. In England and Wales, the highest mortality associated with SARS-CoV-2 has been reported in Black ethnic population, and the lowest in the White population across male and females [6]. Furthermore, BAME individuals are disproportionately represented in the SARS-CoV-2 related mortality rates amongst health and social care workers in the National Health Service (NHS). Of the first 106 deaths amongst NHS staff, 94% of doctors and dentists, 71% of nurses and midwives, and 56% of healthcare support workers were from BAME populations [7]. Given that the NHS workforce is 21% BAME [8], the disproportionate rate of mortality amongst BAME healthcare staff in the UK is alarming. This raises calls to urgently disaggregate all SARS-CoV-2 data by ethnicity, to investigate its implications for the wider population [4].

Ethnicity is complex and is composed of genetic make-up, social constructs, cultural identity, and behavioural patterns [9]. Despite their limitations, ethnic classification systems have been used to explore and predict the health trajectories of different populations [10–12]. Individuals from different ethnic backgrounds vary in behaviours, comorbidities, immune profiles, and risk of infection, as exemplified by the increased morbidity and mortality in BAME populations in previous pandemics [13]. In the 2009 H1N1 pandemic, data from across England reported that BAME individuals experienced an increased risk of death, with the highest risk in the Pakistani sub-group [14]. Furthermore, people living in the highest level of deprivation had a significantly higher risk of death (compared to those in the lowest level of deprivation). The authors concluded that the rapid identification of at-risk groups in the early stages of future pandemics is critical to the implementation of optimal prevention and control measures in vulnerable populations [14].

London, one of most ethnically diverse cities in the UK, has experienced the largest number of SARS-CoV-2 related deaths of any region in the country [15]. The two hospitals included in this study provide health services to seven London boroughs. UK census data [16] reports ethnic diversity across these boroughs as: 63% white and 37% BAME (8.6% black, 16.6% Asian and 12.2% minority ethnicities). In this retrospective analysis we aimed to investigate clinical outcomes by ethnicity in patients hospitalised with SARS-CoV-2.

## Methods

### Study setting

This was a retrospective, observational analysis of patients across two London acute teaching hospitals. The hospitals serve a population of approximately 1.5 million. As well as receiving unwell patients warranting admission, the hospitals adopted a community testing strategy to identify suspected SARS-CoV-2 cases. Unwell patients were screened through 111 call centres and General Practitioners, who referred patients suspected to have SARS-CoV-2, but well enough to be managed in the community to a centralised response team. These patients were followed up via telephone consultation by local surveillance teams and referred for hospital admission in the event of clinical deterioration. This community testing ceased on March 13, 2020, after which only those requiring admission with a compatible clinical syndrome were tested.

### Inclusion and exclusion criteria

All consecutive patients with a real-time polymerase chain reaction (PCR) for SARS-CoV-2 (initially using a proprietary assay run by Public Health England, then from March 10, 2020 onwards a commercial assay from AusDiagnostics®, Australia) admitted to one of the two study sites during March 1 –April 30, 2020 were eligible for inclusion in this study. Demographic data from all patients tested positive for SARS-CoV-2 were analysed for descriptive purposes. Those patients admitted to hospital were included in the study. Patients with no documented ethnicity were excluded.

### Data collection

All tests were processed through one central laboratory and the data available through one electronic healthcare record system (Millennium: Cerner Corporation, Kansas City, Missouri). As part of routine data collection, patients have their demographic data (including age, date of birth, sex, ethnicity and outgoing postcode) recorded from current or previous admissions. This data was extracted for all SARS-CoV-2 positive patients and anonymised for analysis. For patients admitted to hospital, we reviewed the electronic admission records and collected data on the following: comorbidities (using the Charlson Comorbidity Index criteria [17]), admission to the Intensive Care Unit (ICU) and their outcomes (died, discharged alive or transferred to another centre). Length of stay (LOS) was calculated for patients discharged alive or transferred to another centre. Where ethnicity or other demographic data were missing or unclear, the existing patient records were individually reviewed to retrieve the necessary information where possible. Following this reconciliation, patients for whom ethnicity remained unclear were excluded from the analysis.

In order to understand the distribution of social deprivation in the cohort, patients’ outgoing postcodes were analysed using an Indices of Deprivation map (http://dclgapps.communities.gov.uk/imd/iod_index.html). This data were overlapped with the distribution of ethnicity across the boroughs served by the hospitals.

Data was collected from March 14 to June 30, 2020.

### Ethical approval

Data was collected as part of routine care by the responsible clinical team. Data were anonymised at the point of extraction by the study team and no patient identifiable data is reported in this analysis. The study team were not part of the care team. The study protocol was approved by the Antimicrobial Stewardship Group at Chelsea & Westminster NHS Foundation Trust and this was confirmed as a service development by the Research & Development Office of Chelsea & Westminster NHS Foundation Trust. The analysis was conducted in accordance with the Helsinki declaration.

### Study outcome measures

The primary study outcome measure was the rate of admission to ICU and/or mortality in hospitalised patients with a positive diagnosis of SARS-CoV-2 by ethnicity. Secondary outcome measures included analysis of LOS in hospital for those patients who were discharged alive.

### Statistical analysis

The analysis was carried out using the SPSS version 26 (IBM Statistical Product and Service Solutions, IBM Corp., Armonk, N.Y., USA). We utilised chi-square to compare the association between ethnic sub-groups and individual comorbidities. Unadjusted ICU admission and mortality estimates were calculated using univariable binomial logistic regression. Sex, comorbidity and age-adjusted estimates were calculated using multivariable logistic regression. Age and length of stay were normalised by calculating their fractional ranks and using an inverse density function as both were skewed in their distributions. Age was also fitted as a categorical variable in the multivariable models. The ages of the hospitalised white and BAME populations were compared with an independent samples t-test. A further t-test compared the ages of the cohort which died amongst these ethnic groups. To compare the effect of ethnicity on length of stay whilst controlling for age, sex and comorbidities, a one-way analysis of co-variance (ANCOVA) was conducted. Levene’s test for equality of variance and normality checks were carried out and assumptions met. Statistical significance was defined as a *p* ≤ 0.05.

## Results

Between March 1 –April 30, 2020 1104 patients across the two sites were diagnosed with SARS-CoV-2 following a positive PCR test result. In total 847 were admitted to hospital and 257 were managed in the community. Ethnicity was not stated in the records of 202/847 (23.8%) hospitalised patients and 84/257 (32.7%) of community managed patients. The final analysis therefore included 645 hospitalised and 173 community managed patients.

### Description of cohort

The male to female ratio amongst all patients was 60:40 (487 and 331, respectively). The mean age was 62 years with a range of 1 week– 106 years. Of the 818 patients, 408 (49.9%) were white and 410 (50.1%) were BAME. The BAME sub-groups were as follows: 75/410 (18.3%) black, 184/410 (44.9%) Asian, 136/410 (33.2%) minority ethnicities, and 15/410 (3.7%) mixed ethnicities. Table 1 summarises the breakdown of ethnicity, sex and age in the community managed and hospitalised patient populations.

**Table 1:** Demographics of SARS-CoV-2 positive patients in a Central London population (March 1 –April 30, 2020).

		Total	Community	Hospitalised
**Number of patients (N)**		818	173	645
**Male (%)**		487 (59.5)	101 (58.4)	386 (59.8)
**Age**	Mean (SD)	61.5 (21)	46.5 (20.2)	65.5 (19.3)
	Median (IQR)	64 (31)	43 (31)	68 (26)
	Range (Min–Max)	0–106	0–93	0–106
**Ethnicity (%)**	White	408 (49.9)	88 (50.9)	320 (49.6)
	BAME	410 (50.1)	85 (49.1)	325 (50.4)
	Black	75 (9.2)	9 (5.2)	66 (10.2)
	Asian	184 (22.5)	42 (24.3)	142 (22)
	Minority ethnicities	136 (16.6)	28 (16.2)	108 (16.7)
	Mixed ethnicity	15 (1.8)	6 (3.5)	9 (1.4)

**Fig 1:**
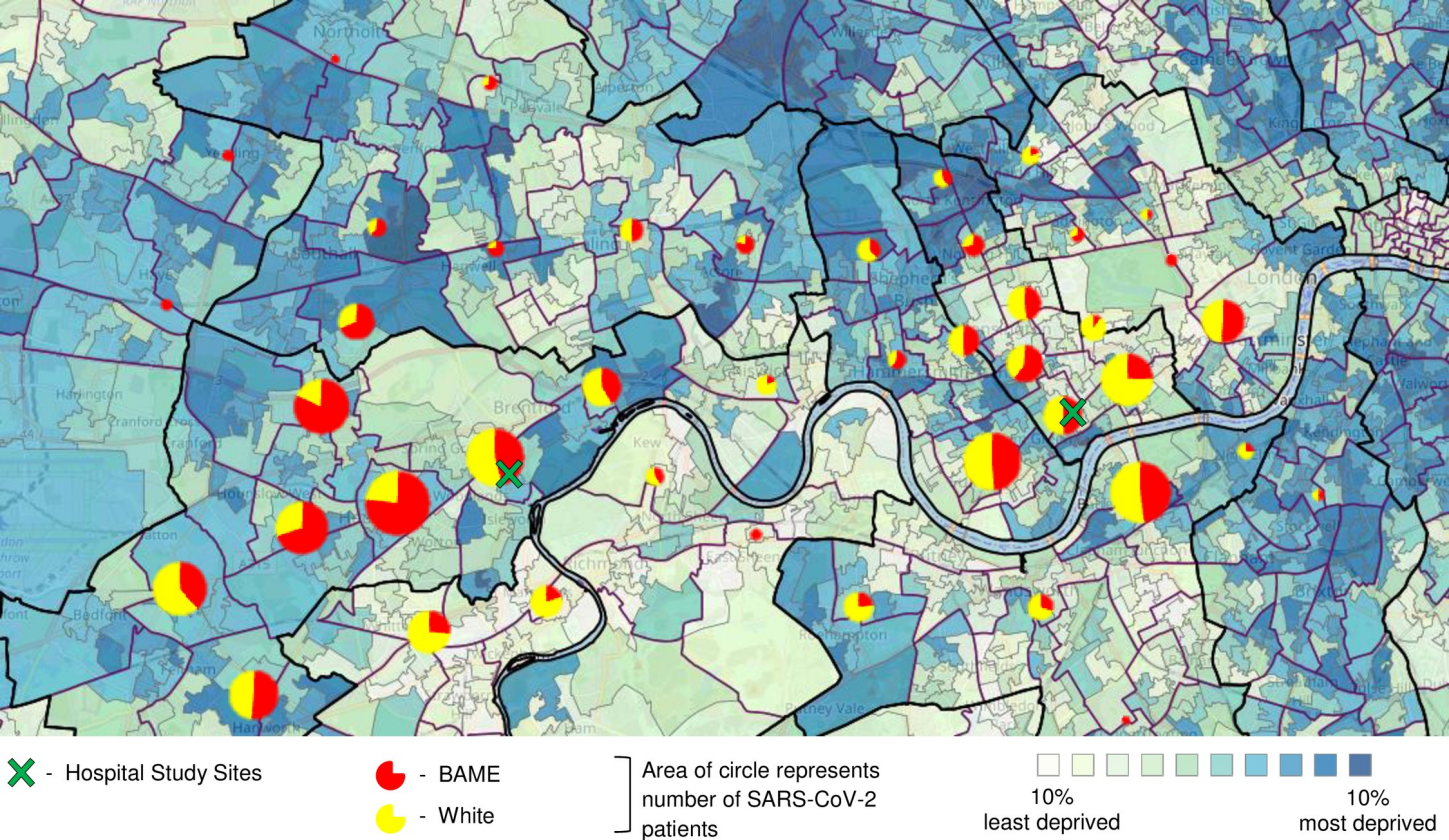
Geographic distribution of SARS-CoV-2 positive patients against areas of social deprivation, across a Central London population (March 1 –April 30, 2020). Indices of Deprivation: 2019 map. http://dclgapps.communities.gov.uk/imd/iod_index.html.

Fig 1 represents the distribution of patients by ethnicity (defined as white vs BAME) against a map of social deprivation in the boroughs served by the hospitals. Both hospitals serve boroughs with ethnically diverse populations, and a varied social deprivation profile.

### Hospital cohort

The male to female ratio of hospitalised patients was 60:40 in both white and BAME populations (Table 2). There was a statistically significant difference between the mean age at admission in the white population (69.30 years, 95% CI 67.17–71.43) and the BAME population (61.70 years, 95% CI 59.70–63.73) (p<0.001). There was a statistically significant difference in the proportions of diabetes (***χ***^**2**^ = 22.11, p<0.001), dementia (***χ***^**2**^ = 13.06, p = 0.011) and malignancy (***χ***^**2**^ = 14.76, p = 0.005) between ethnic sub-groups.

**Table 2:** Demographics and comorbidities by ethnicity for hospitalised SARS-CoV-2 positive patients from two London hospitals (March 1 –April 30, 2020).

Variable	Total	White	Black	Asian	Minority ethnicities	Mixed ethnicity	P value
	645	*320	66	142	108	9	
**Demographics n(%)**							
**Male (%)**	386 (59.8)	191 (59.7)	42 (63.6)	82 (57.7)	64 (59.3)	7 (77.8)	
**Mean age at admission**		69.3	62.2	57.7	58.3	53	
**Age categories**							
<17	14	6 (42.9)	2 (14.3)	3 (21.4)	2 (14.3)	1 (7.1)	**<0.001**
18–39	48	21 (43.8)	1 (2.1)	9 (18.8)	16 (33.3)	1 (2.1)	
40–49	57	19 (33.3)	10 (17.5)	14 (24.6)	12 (21.1)	2 (3.5)	
50–59	100	36(36)	14 (14)	21 (21)	27 (27)	2 (2)	
60–69	114	47 (41.2)	19 (16.7)	29 (25.4)	19 (16.7)	0 (0)	
70–79	141	78 (55.3)	10 (7.1)	36 (25.5)	16 (11.3)	1 (0.7)	
>80	171	113 (66.1)	10 (5.8)	30 (17.5)	16 (9.4)	2 (1.2)	
**Comorbidities n(%)**							
Hypertension	297	148 (46.3)	35 (53)	70 (49.3)	41 (38)	3 (33)	0.26
Cardiac disease	150	80 (25)	7 (10.6)	38 (26.8)	23 (21.3)	2 (22.2)	0.14
Chronic lung disease	130	72 (22.5)	15 (22.7)	25 (17.6)	17 (15.7)	1 (11.1)	0.45
Diabetes Mellitus	199	76 (23.8)	27 (40.9)	61 (43.0)	34 (31.5)	1 (11.1)	**<0.001**
Chronic liver disease	11	5 (1.6)	1 (1.5)	2 (1.4)	3 (2.8)	0	0.91
Chronic kidney disease	83	43 (13.4)	10 (15.2)	22 (15.5)	7 (6.5)	1 (11.1)	0.27
Obesity	33	17 (5.3)	6 (9.1)	4 (2.8)	6 (5.6)	0	0.37
Chronic neurological disease	48	28 (8.8)	4 (6.1)	6 (4.2)	10 (9.3)	0	0.36
Dementia	51	37 (11.6)	2 (3.0)	8 (5.6)	3 (2.8)	1 (11.1)	**0.011**
Malignancy	52	34 (10.6)	8 (12.1)	10 (7.0)	0	0	**0.005**

* p-values represent significance of univariable association between the variable and ethnic sub-groups (white, black, Asian, minor ethnicities and mixed ethnicities).

### Length of stay

LOS analysis excluded readmissions (10 patients) as their total LOS was interrupted. The median LOS for the remaining patients was 8 days (IQR 4–16), (Table 3). There was no statistically significant difference in LOS between white and BAME groups (p = 0.026).

**Table 3:** Outcomes by ethnicity for hospitalised SARS-CoV-2 positive patients from two London hospitals (March 1 –April 30, 2020).

Outcomes	Total	White	Black	Asian	Minority ethnicities	Mixed ethnicity
**Length of Stay**						
Median (IQR)	8 (12)	9 (14)	8 (12)	6 (6)	8 (11)	5 (42)
[Min- max]	[0–86]	[0–88]	[0–43]	[0–86]	[1–65]	[2–59]
**ICU admission**	86 (13.3)	31 (9.7)	12 (18.2)	20 (14.1)	21 (19.4)	2 (22.2)
**Discharged alive**	401 (62.2)	198 (61.9)	40 (62.1)	75 (52.8)	81 (75)	6 (66.7)
**Mortality in admission episode**	225 (34.9)	112 (35.0)	23 (34.8)	64 (45.1)	24 (22.2)	2 (22.2)
**Transferred**	13 (2.0)	7 (2.2)	2 (3)	1 (0.7)	2 (1.9)	1 (11.1)
**Currently inpatient**	6 (0.9)	3 (0.9)	0 (0)	2 (1.4)	1 (0.9)	0 (0)

### ICU admission

The male to female ratio of patients admitted to ICU in the white population was 66:34 versus 71:29 in BAME populations. The mean age was 57 years in the white population versus 56 years in the BAME population.

The proportion of admission to ICU for all patients was 13.3%. The proportion of admission to ICU by ethnic sub-group was as follows: white 9.7%, black 18.2%, Asian 14.1%, minority ethnicities 19.4% and mixed ethnicities 22.2% (Table 3).

Unadjusted analysis of ethnicity as a predictor of ICU admissions revealed that BAME populations were at greater odds of admission compared to the white population (OR 1.90: 95% CI 1.19–3.04, p = 0.008) (Table 4). In the multivariable model, when adjusted for age, sex and comorbidities, the BAME population was not significantly more likely to have an ICU admission.

**Table 4:** Multiple logistic regression analysis of the association between ethnicity and ICU admission in SARS-CoV-2 positive patients in two London hospitals, adjusted for sex, age and comorbidities (March 1 –April 30, 2020).

ICU Admission n = 86					
		Unadjusted		Adjusted	
		OR (95% CI)	P value	OR (95% CI)	P value
**Ethnicity**	White	**	–	–	–
	Black	2.07 (1.00–4.29)	**0.050**	1.45 (0.64–3.29)	0.37
	Asian	1.53 (0.84–2.79)	0.166	1.48 (0.76–2.89)	0.26
	Minority Ethnicities	2.25 (1.23–4.12)	**0.008**	1.47 (0.75–2.89)	0.26
	Mixed Ethnicities	2.66 (0.53–13.39)	0.234	2.671 (0.44–16.06)	0.28
**Age**	<17	–	–	0.000	0.99
	18–39	–	–	1.35 (0.468–3.861)	0.58
	40–49	–	–	***	–
	50–59	–	–	2.53 (1.049–6.085)	**0.04**
	60–69	–	–	2.46 (0.998–6.041)	**0.05**
	70–79	–	–	0.60 (0.201–1.807)	0.37
	>80	–	–	0.13 (0.025–0.693)	**0.02**
Sex*		–	–	1.61 (0.93–2.81)	0.91
**Comorbidities**	Hypertension	–	–	1.26 (0.689–2.294)	0.46
	Cardiac disease	–	–	0.66 (0.274–1.587)	0.35
	Chronic lung disease	–	–	1.33 (0.692–2.546)	0.39
	Diabetes Mellitus	–	–	0.58 (0.306–1.093)	0.09
	Chronic liver disease	–	–	0.89 (0.095–8.381)	0.92
	Chronic kidney disease	–	–	0.11 (0.014–0.833)	**0.03**
	Obesity	–	–	2.77 (1.183–6.491)	**0.02**
	Chronic Neurological disease	–	–	0.34 (0.098–1.211)	**0.0097**
	Dementia	–	–	0.000	0.99
	Malignancy	–	–	0.39 (0.083–1.798)	0.23

* Odds ratios are given with females as the reference group.

** Odds ratios are given with the white population as the reference group.

*** Odds ratios are given with the 40–49 years group as the reference group.

Unadjusted analysis of the association between ethnic sub-groups and ICU admission showed that black and minority ethnic groups had higher odds of admission to ICU (OR 2.07: 95% CI 1.0–4.29, p = 0.05 and OR 2.25: 95% CI 1.23–4.12, p = 0.08 respectively). However, when adjusting for age, sex and comorbidities, no ethnic sub–group was significantly more likely to have an ICU admission.

### Mortality

The inpatient mortality was 225/645 (34.9%) (Table 3). The proportion of mortality in the white and BAME populations were 35% and 34.8%, respectively. Among those who died, there was a statistically significant difference between the mean age in the white population (77.4 years, 95% CI 76.1–78.70) and the BAME population (71.44 years, 95% CI 69.90–72.90) (p<0.001).

Unadjusted analysis of ethnicity as a predictor of mortality revealed that BAME patients did not have higher odds of death compared to the white population (OR 0.99: 95% CI 0.72–1.37, p = 0.99) (Table 5). This remained the case once adjusted for age, sex and comorbidities (OR 1.37: 95% CI 0.92–2.03, p = 0.12). Following adjustment, male patients were shown to be significantly more likely to die (OR 1.75: 1.18–2.60, p = 0.004).

**Table 5:** Multiple logistic regression analysis of the association between ethnicity mortality in SARS-CoV-2 positive patients in two London hospitals, adjusted for sex and age (March 1 –April 30, 2020).

Mortality n = 225					
		Unadjusted		Adjusted	
		OR (95% CI)	P value	OR (95% CI)	P value
**Ethnicity**	White	**	–	**	–
	Black	0.99 (0.57–1.73)	0.98	1.32 (0.68–2.57)	0.41
	Asian	1.52 (1.02–2.28)	**0.04**	1.99 (1.22–3.25)	**0.006**
	Minority Ethnicities	0.53 (0.32–0.88)	**0.02**	0.79 (0.43–1.43)	0.43
	Mixed Ethnicities	0.53 (0.11–2.60)	0.43	0.91 (0.12–6.74)	0.93
**Age**	<17	–	–	2.29 (0.350–10.151)	0.49
	18–39	–	–	1.88 (0.350–10.151)	0.461
	40–49	–	–	***	–
	50–59	–	–	3.39 (0.919–12.532)	0.067
	60–69	–	–	7.41 (2.082–26.350)	**0.002**
	70–79	–	–	11.77 (3.303–41.932)	**<0.001**
	>80	–	–	12.16 (3.361–44.011)	**<0.001**
Sex*		–	–	1.79 (1.204–2.672)	**0.004**
**Comorbidities**	Hypertension	–	–	1.54 (1.016–2.318)	0.042
	Cardiac disease	–	–	2.11 (1.343–3.3–6)	**<0.001**
	Chronic lung disease	–	–	1.22 (0.771–1.915)	0.40
	Diabetes Mellitus	–	–	1.29 (0.847–1.969)	0.23
	Chronic liver disease	–	–	2.88 (0.603–13.757)	0.19
	Chronic kidney disease	–	–	2.29 (1.304–4.012)	**0.004**
	Obesity	–	–	4.05 (1.710–9.610)	**<0.001**
	Chronic Neurological disease	–	–	1.35 (0.686–2.666)	0.38
	Dementia	–	–	1.07 (0.547–2.084)	0.85
	Malignancy	–	–	1.36 (0.712–2.588)	0.35

* Odds ratios are given with females as the reference group.

** Odds ratios are given with the white population as the reference group.

*** Odds ratios are given with the 40–49 years group as the reference group.

Unadjusted analysis by ethnic sub-groups showed that the Asian population had greater odds of death (OR 1.52: 95% CI 1.02–2.28, p = 0.04) whereas minority ethnicities had decreased odds of death (OR 0.53: 95% CI 0.32–0.88, p = 0.015). After adjusting for age, sex and comorbidities, the Asian population remained significantly more likely to die (OR 1.99: 95% CI 1.22–3.25, p = 0.06). Minority ethnicities were, however, shown to be at no increased odds of death (OR 0.79: 95% CI 0.43–1.43, p = 0.43).

## Discussion

Amongst the hospitalised patients, we found significant differences in demographic data and outcomes between the ethnic groups. Notably, the BAME population were younger at both admission and at death when compared to the white population. There did not appear to be a difference in ICU admission amongst the ethnic groups when adjusting for age, sex and comorbidities. In keeping with existing evidence, we found increasing age and male sex to be associated with higher odds of death [1].

Although the mortality between white and BAME populations was similar, once adjusted for age, sex and comorbidities, we found higher odds of death amongst the Asian patient population. Whilst Asian patients are more likely die, this is not reflected by the ICU admission rates in this cohort, which is often considered a surrogate marker of critical illness. This may be due to physiological factors such as increased comorbidity burden, leading to earlier palliative decisions, and patient factors such as delayed hospital presentation, leading to patients presenting much further along the natural history of the disease. This may preclude these groups from critical care. Social and cultural factors such as family and support networks may affect healthcare accessibility which may also impact outcomes. However, a disproportionate rate of mortality has been reported amongst healthcare workers [7], suggesting that ethnicity is a wider determinant of health beyond social inequality. The disparity between mortality rate and ICU admission needs to be further investigated. This highlights the need to take a broader and more inclusive approach to studying the role of ethnicity as part of wider socio-cultural determinants of health.

**Fig 2:**
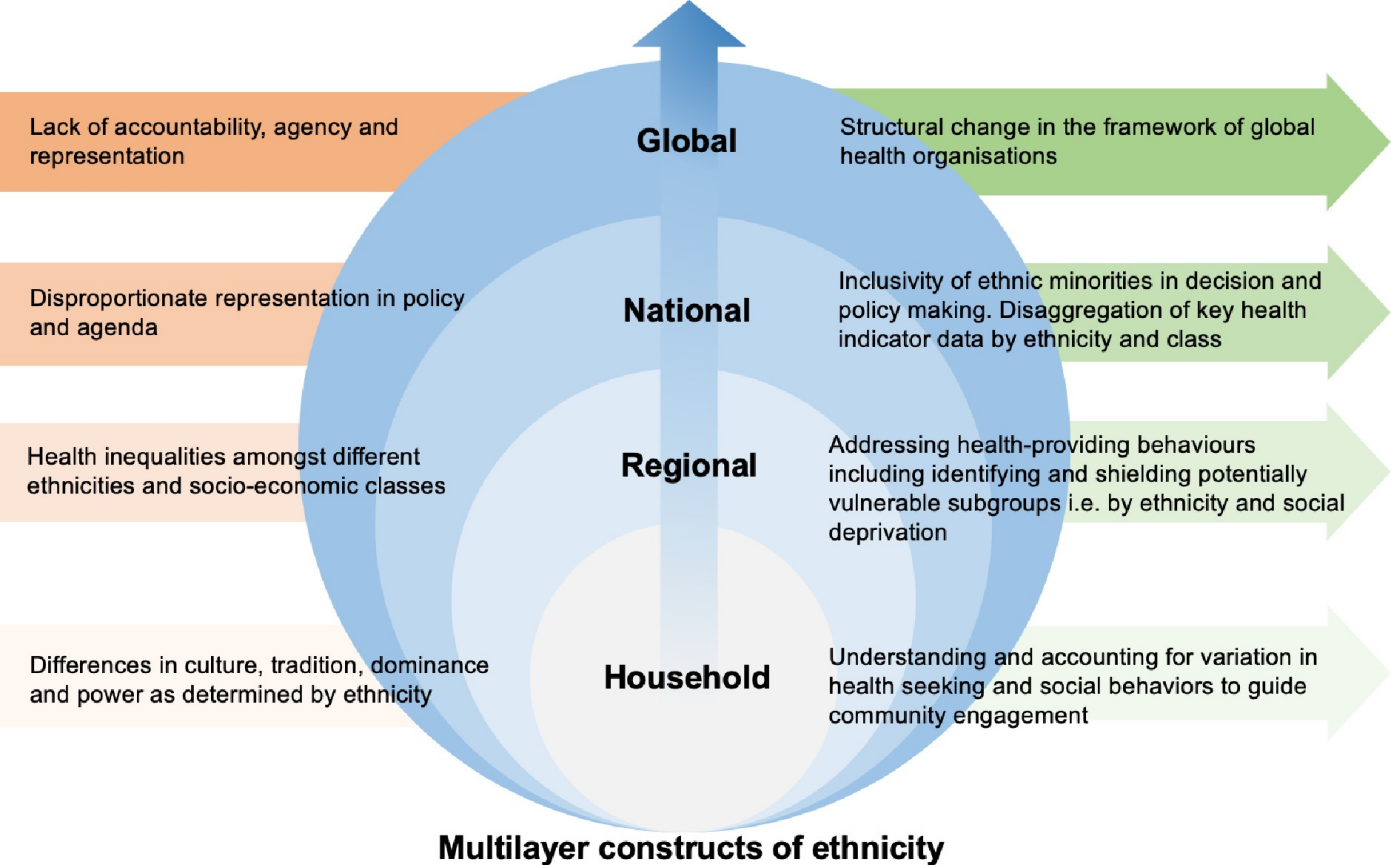
The current gaps and potential solutions in how ethnicity acts as a wider social determinant of health.

The emerging evidence from the limited clinical studies that have data on the ethnicity of patients with COVID-19 does suggest that BAME populations are at greater risk of acquiring SARS-CoV-2 infection, as well as having poorer COVID-19 clinical outcomes [18]. Disaggregating population level data by ethnicity will provide an opportunity for large scale analyses of the impact of ethnicity on COVID-19 clinical outcomes across broad populations with different socio-demographic profiles. This is critical as we need to go beyond sex and gender when studying diseases in populations, and consider the vulnerabilities and advantages that are related to social factors such as ethnicity, migrant status, geographical location, disabilities and health status [19]. This will enable the prediction and preparedness of healthcare needs for sub-populations, helping address health inequalities. Addressing health inequalities at all societal levels is paramount to developing targeted healthcare strategies from an individual to a global level (Fig 2). This hinges on understanding how human health is shaped by the interaction of different social constructs and conditions (i.e. ethnicity, sex, socio-economic class, and geography) which in turn interact within societal structures e.g. laws, policies, and media [20].To fully understand the role of ethnicity on health outcomes, sophisticated analysis which takes into consideration how the connected and complex systems of health, policy, and society are interlinked is required. In addition to age, gender, ethnicity, and comorbidity, other stressors from global and national to household factors, influence the constructs of ethnicity and how it intersects with health as we have observed in this pandemic. The SARS-CoV-2 pandemic has demonstrated how race, class and gender intersect with health resulting in significant disparities in outcomes. These intersecting stressors and social determinants of health need to be investigated if we are to understand how, and why race, class and gender affect behavioural and health outcomes. The skewed statistics in the mortality of healthcare professionals from black, Asian, and minority ethnic backgrounds cannot be explained away by only treating ethnicity as a social determinant. Political and structural investment in ensuring inclusivity, agency and representation of diverse ethnic groups is needed if we are to positively influence the social constructs of ethnicity. Finally, factors contributing towards health-seeking and health-providing behaviours need to be identified to help guide public health interventions.

As in other pandemics, the severity of the risk of SARS-CoV-2 varies widely depending not only on the physiological but also socio-economic factors [14]. Recognising these complex dynamics will allow for a better understanding of social inequalities as well as the drivers for these inequalities, allowing researchers, public health policy makers and governments to respond to them [21]. The UK is easing the national lockdown. With the emerging evidence that there is a subset of the population at higher risk of poorer outcomes and limited knowledge of why this may be the case, targeted and more sophisticated public health strategies are needed.

### Study limitations

The analysis excluded patients for whom ethnicity was unknown (25% of the cohort), as we could not assume this data was missing at random. Decisions to triage patients to the ICU are determined by non-patient factors such as bed capacity, variation in escalation protocols, and patient and family factors. It was not possible to retrospectively collect these data, however, ICU bed capacity was not breached during the study period meaning there was no need to prioritise patients going to ICU. Smoking status was missing for the majority of patients and since the study sample size was not large enough, we excluded smoking status as a variable in the analysis. The two areas served by the hospitals in this study had contrasting wealth indices and ethnic diversities [14]. We used postcode data to visually illustrate the distribution of cases by ethnicity and social deprivation. Whilst this provides an overview, this could not be contextualized, as documented social deprivation indices are based on Lower Layer Super Output Areas (a geospatial statistical unit used in England and Wales to facilitate the reporting of small area statistics). NHS patient data is not classified to this level, which would be needed for an accurate analysis.

## Conclusions

Admission and death were observed at a younger age in BAME patients with SARS-CoV-2 relative to white patients. Despite this, BAME populations had similar rates of admission to ICU compared to the white population. The reason for these disparities is not fully understood and needs to be addressed. Investigating ethnicity as a clinical risk factor remains a high public health priority; however, future work must also consider it as part of the wider socio-cultural determinant of health.
